# Fifty percent of patients undergoing periacetabular osteotomy for hip dysplasia showed normal findings upon neonatal ultrasound screening: Symptomatic hip dysplasia in adulthood despite inconspicuous neonatal hip ultrasound

**DOI:** 10.1007/s00132-023-04357-7

**Published:** 2023-03-17

**Authors:** Marco Haertlé, Harun Hawi, Henning Windhagen, Nils Becker, Sufian S. Ahmad

**Affiliations:** Klinik für Orthopädie der Medizinischen Hochschule Hannover im Annastift, Anna-von-Borriesstr. 1–7, 30625 Hannover, Germany

**Keywords:** Infant hip sonography, Acetabulum, Reorientation osteotomy, Developmental hip dysplasia, Secondary hip osteoarthritis, Sonographie der Säuglingshüfte, Acetabulum, Periacetabuläre Osteotomie, Entwicklungsbedingte Hüftdysplasie, Sekundäre Coxarthrose

## Abstract

Developmental dysplasia of the hip (DDH) is characterized by the pathomorphology of inadequate acetabular coverage of the femoral head leading to increased loading of the articular surface and acetabular rim. If left untreated, this ultimately leads to osteoarthritis. Germany introduced a nationwide universal ultrasound screening program for all newborn infants in 1996. Subsequently, the incidence of undiagnosed hip dislocation was significantly reduced. In this consecutive series of patients who underwent periacetabular osteotomy for the treatment of symptomatic dysplasia of the hip between October 2014 and October 2022 data regarding the U3 screening examination were analyzed. The data included whether the examination was performed, whether the findings were positive or negative, whether the patients underwent any form of treatment in the case of a positive finding and whether a control X‑ray was performed. This study provides evidence that acetabular undercoverage cannot be ruled out based on a normal finding in ultrasonography screening. Furthermore, the study also shows that residual dysplasia may persist despite attempts of conservative treatment.

**Graphic abstract:**

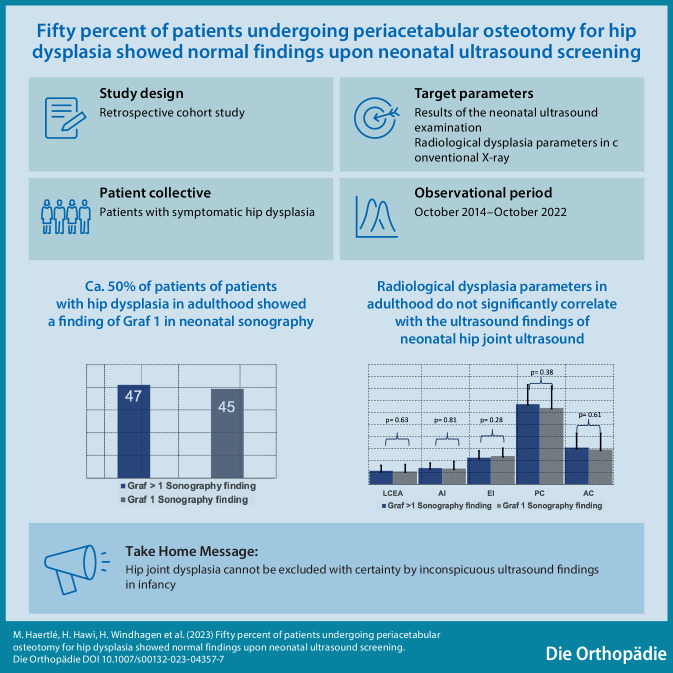

## Abstract (de)

Das Krankheitsbild der entwicklungsbedingten Hüftdysplasie („developmental dysplasia of the hip“, DDH) ist pathomorphologisch charakterisiert durch eine unzureichende Überdachung des Hüftkopfes durch das Acetabulum. Diese Inkongruenz der Artikulation führt zu einer erhöhten Belastung der Gelenkflächen sowie des Pfannenrandes und unbehandelt letztendlich zu einer Arthrose des Hüftgelenks. In Deutschland wurde 1996 ein flächendeckendes Ultraschallscreening der Hüftgelenke für alle Neugeborenen eingeführt. Daraufhin sank die Zahl der nicht diagnostizierten Hüftluxationen signifikant. In dieser konsekutiven Serie von Patienten, bei denen zwischen Oktober 2014 und Oktober 2022 eine periacetabuläre Osteotomie zur Behandlung einer symptomatischen Hüftdysplasie durchgeführt worden war, wurden die Daten zur U3-Vorsorgeuntersuchung analysiert. Unter anderem wurde erfasst, ob die Untersuchung durchgeführt wurde, ob der Befund regelhaft oder pathologisch war, ob die Patienten im Falle eines pathologischen Befundes in irgendeiner Form behandelt wurden und ob ein Kontrollröntgenbild angefertigt wurde. Diese Studie erbringt die Evidenz dafür, dass ein Defizit der acetabulären Überdachung des Hüftgelenks aufgrund eines regelhaften Befundes im Rahmen des Ultraschallscreenings nicht ausgeschlossen werden kann. Darüber hinaus verdeutlicht die Studie auch, dass trotz konservativer Behandlungsversuche eine Restdysplasie bestehen bleiben kann.

A universal nationwide ultrasound screening program was introduced in Germany in 1996 to reduce the incidence of developmental dysplasia of the hip (DDH). Despite the introduction of this program, a rise in young adults with symptomatic acetabular undercoverage seeking treatment in hip preservation centers is being seen. This study shows that acetabular undercoverage cannot be ruled out based on a normal ultrasound finding at neonatal screening.

## Introduction

Developmental dysplasia of the hip (DDH) is characterized by the pathomorphology of insufficient acetabular coverage of the femoral head, resulting in instability and increased loading of the articular surface and acetabular rim. These pathological loads on the articular cartilage, if left undetected or untreated, lead to intra-articular structural damage and subsequently to the development of secondary osteoarthritis of the hip joint. It is estimated that approximately 20–40% of all cases of adult hip osteoarthritis are due to unrecognized or untreated DDH [1]. The prevalence of DDH in the population can range from 1% to 40% depending on a multitude of factors including ethnicity, gender, pregnancy-related aspects and radiographic thresholds of definition [2–5]**. **In general, a lateral central edge angle (LCEA) of < 20°, an acetabular index (AI) > 15°, and a femoral head extrusion index (EI) > 25% on a mature anteroposterior (AP) pelvic radiograph are signs of undercoverage in terms of DDH [6, 7]. If insufficient acetabular roofing is detected in early childhood, it can be often addressed with non-surgical methods of splinting [8]. In skeletally mature patients, reorientation osteotomy represents the gold standard for treatment of the underlying pathomorphology. The two most prominent types of osteotomy that are commonly performed in adults include the triple Tönnis osteotomy and the periacetabular Ganz osteotomy (PAO) [9, 10].

It has been well described that DDH is frequently abundant despite the absence of common risk factors, making selective screening difficult [11]. Therefore, Germany introduced a nationwide ultrasound screening program in 1996 for all newborn infants using the Graf method. This procedure is commonly referred to as the U3 check-up examination [12, 13].

The effectiveness of ultrasonography as a preventive measure is determined by the subsequent need for a treatment intervention on detection of DDH. Several studies underline the potential of universal ultrasound screening as a successful and cost-efficient preventive tool [14, 15]. Von Kries et al. demonstrated that introduction of sonography as part of the screening examination in Germany significantly reduced the need for surgical interventions in children up to 5 years of age [16]. The examiner evaluates the bony and cartilaginous roof of the acetabulum by measuring the alpha and beta angles and describes the position of the femoral head within the acetabulum.

Despite the introduction of screening programs, there has been a rise in young adults with symptomatic acetabular undercoverage seeking treatment in hip preservation centers. This is accompanying increased awareness and the recent emergence of the field of hip preservation surgery.

It was the aim of this study to focus on a consecutive cohort of patients undergoing PAO surgery for symptomatic hip dysplasia. It was hypothesized that patients undergoing PAO surgery showed positive ultrasonography findings as infants.

## Methods

A consecutive series of patients who underwent periacetabular osteotomy for the treatment of symptomatic dysplasia of the hip between October 2014 and October 2022 were considered for inclusion in the study. Inclusion criteria were symptomatic hip dysplasia that was diagnosed based on an anteroposterior (AP) pelvic radiograph. All radiographs were obtained in a standardized manner as previously described by Tannast et al. [7] and three radiographic findings were necessary for the diagnosis of dysplasia, a lateral center edge angle (LCEA) of ≤ 20°, anterior wall coverage (AC) of < 15° and an acetabular extrusion index (EI) of > 25%. Patients fulfilling these criteria and undergoing PAO were considered eligible for inclusion in the study. Patients who underwent PAO for acetabular retroversion, posterior dysplasia, or for deformities in association with neuromuscular diseases, were excluded to ensure the homogeneity of the cohort.

After obtaining formal consent for anonymous analysis of data, patients were contacted for retrieval of a simple set of data regarding the U3 screening examination including whether the examination was performed, whether the findings were positive or negative, whether the patients underwent any form of treatment in the case of a positive finding and whether a control X‑ray was performed.

**Fig. 1:**
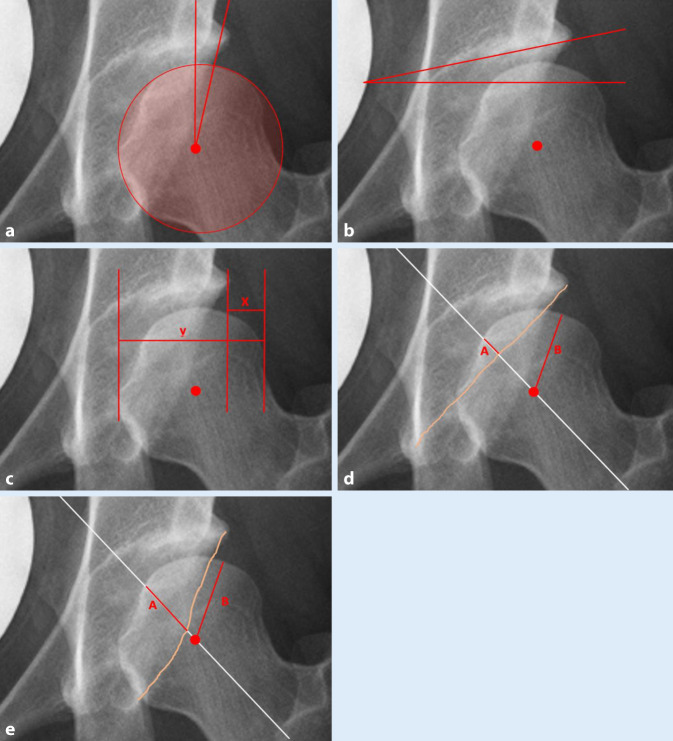
Anteroposterior (AP) pelvic radiographs of the radiological measurement in native pelvic X‑ray obtained in a standardized manner as previously described by Tannast et al. **a** Lateral center edge angle, **b** acetabular index, **c** extrusion index (x /y ×100), **d** anterior coverage (A /B ×100) and **e** posterior coverage (A /B ×100). *Red dot* femoral head center, *white line* axis of the femoral neck, *pink line* rim of the anterior or posterior acetabulum

Demographic data of all patients were tabulated. In addition to the abovementioned radiographic measures that were used for surgical indications, the acetabular index and posterior wall coverage were also measured to enable a global depiction of acetabular coverage (Fig. 1).

Due to the retrospective nature and inhomogeneity of reporting, the results were documented based on a binary scale of either Graf I normal finding or Graf II–IV abnormal finding requiring a Pavlic harness, splinting or surgery.

Comparison between groups was performed using an unpaired t test. A normal distribution was assumed. Values were expressed as mean and standard deviation. Positive predictive values were calculated. *P*-values of < 0.05 were considered significant. Statistical analysis was performed using IBM® SPSS® Statistics software version 28.0.1.1 (14) (64 bit version, IBM, Chicago, IL, USA) and Microsoft® Excel version 16.43 (Microsoft, Redmond, WA, USA).

## Results

**Fig. 2:**
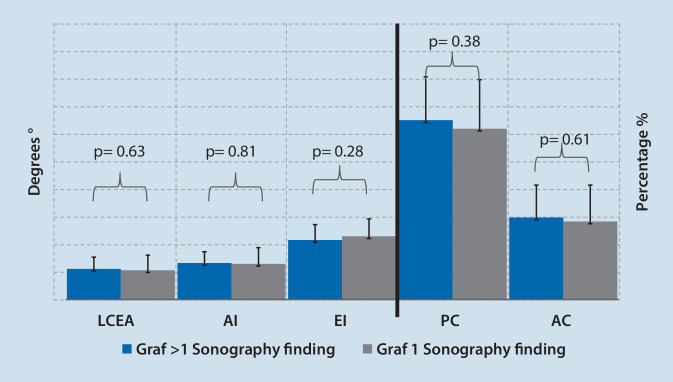
Comparison of radiological parameters in the mature pelvis in AP projection showed no significant difference between patients presenting with sonographic hip development Graf 1 or Graf > 1 in U3 examination. *LCEA* lateral center edge angle, *AI* acetabular index, *EI* extrusion index, *PC* posterior coverage, *AC* anterior coverage

**Fig. 3:**
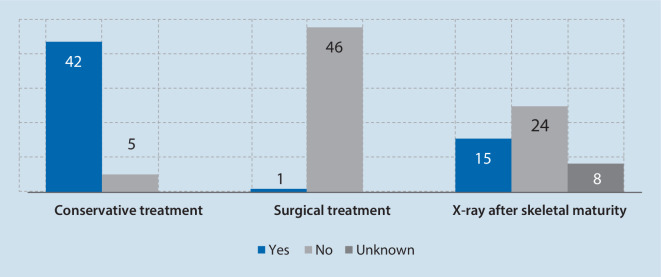
Selected treatment in patients with positive findings in neonatal hip sonography (Graf > 1) as well as rate of radiographic follow-up in patients with positive findings in neonatal hip sonography

From a total of 134 patients, 92 individual patients managed to provide a conclusive result of their infant screening. Of these, 82 (89.1%) patients were females and 10 (10.9%) males. The mean age at time of surgery was 27.4 years (standard deviation, SD±7.3 years, range 14.6–41.5 years). The mean acetabular morphometric measures prior to PAO were: LCEA 13.5° (SD±6.1°, range −6.2–24.5°), AI 16.3° (SD±6.3°, range 6.3–42.1°), EI 27.7% (SD±7.4%, range 12.0–56.0%), AC 36.2% (SD±15.5%, range 0.0–71.0%) and PC 79.3% (±21.0%, range 0.0–124.0%) (Fig. 2). All patients underwent U3 ultrasonography screening of the hip as infants. In 47 cases (51.1%) ultrasonography revealed a positive finding (Graf > 1) and 45 (48.9%) hips were reported to be normal (Graf 1) at U3 and needed to undergo pelvic osteotomy in adulthood due to hip dysplasia. The true positive rate (sensitivity) of ultrasonography was calculated to be 51.0%. Both patient groups with DDH detection and without DDH detection in the U3 examination, did not differ regarding gender distribution, time point of surgery and radiographic acetabular measurements (Table 1, Fig. 1, *p* > 0.05). Figure 3 shows the rate and distribution of treatment in patients with positive findings and depicts whether a pelvic X‑ray was performed after skeletal maturity.

**Table 1:** Characteristics of adult patients stratified based on either a positive or negative screening history upon neonatal U3 ultrasound examination

	Positive DDH at U3 (*n* = 47)	Negative DDH at U3 (*n* = 45)	*p*-value
Age (years, pelvic osteotomy)	28.3 ± 7.6	26.3 ± 7.0	0.36
Gender (female/male)	43 f/4 m	39 f/6 m	0.46
LCEA (°±SD)	13.8 ± 5.4	13.2 ± 6.9	0.63
AI (°±SD)	16.5 ± 5.1	16.1 ± 7.4	0.81
EI (%±SD)	26.9 ± 7.0	28.6 ± 7.9	0.28
PC (%±SD)	81.2 ± 19.7	77.3 ± 22.3	0.38
AC (%±SD)	37.1 ± 14.7	35.3 ± 16.5	0.61

*DDH *developmental dysplasia of the hip, *LCEA* lateral center edge angle, *AI* acetabular index, *EI* extrusion index, *PC* posterior coverage, *AC* anterior coverage, *f* female, *m* male, *SD* standard deviation

## Discussion

The most important finding of this study was that 50% of young adults undergoing periacetabular osteotomy for DDH in a German institution reported a nonpathological neonatal hip sonography scan result. The low sensitivity of 50% underlines a high likelihood of misdiagnosing a hip that is likely to develop symptoms due to acetabular coverage.

Reports of this type are lacking in the literature. This is due to the concept of this study that was intended to put the focus on the morphology of acetabular undercoverage after skeletal maturity.

There have been reports looking into the diagnostic and screening value of sonography in preventing treatment in infants and toddlers. Ihme et al. found that on average 0.26 of 1000 infants received treatment for DDH at the age of 10 months to 5 years despite unremarkable sonographic screening of the hip [12]. A slightly higher incidence rate of 0.74 per 1000 births was described by Clarke et al. for selective DDH sonography screening [17].

The problematic issue of technical mistakes and misinterpretation of anatomic structures during hip sonography have previously been outlined [18]. The German Association of Statutory Health Insurance Physicians reported that 32% of consultants who applied for initial certification in infant hip sonography did not undertake adequate training [19]. Bucher et al. reported that the initial diagnosis at neonatal sonography screening of 132 neonates was confirmed by a certified pediatrician in only 75 cases [20].

It has been shown that the successful implementation of newborn screening was accompanied by a subsequent reduction in the rate of open surgery. This is likely due to the reduced incidence of neglected hip dislocation; however, the prevalence of acetabular undercoverage in the adult population remains high with late onset of symptoms at various stages of life depending on the severity of disease [12, 21, 22]. This form of DDH is also known as adolescent-onset acetabular dysplasia and is commonly associated with a greatly reduced hip and acetabular undercoverage. The causes of late-onset acetabular dysplasia are not fully understood. A possible explanation was described by Tönnis and Remus in a case series, demonstrating irregular calcification and structural irregularities of the triradiate cartilages in the acetabular roof and superior acetabular rim in patients with adolescent-onset acetabular dysplasia [23]. The extent to which further radiological screening is necessary after skeletal maturity should be assessed in the presence of symptoms or risk factors. The high prevalence and consequences of late-onset acetabular dysplasia have been underlined in arthroplasty registries. It was shown that one third of arthritic hips undergoing hip replacement surgery before the age of 60 years showed signs of acetabular undercoverage [24].

Based on the results of the current study, half of all patients undergoing pelvic osteotomy had some form of conservative treatment due to a positive sonography finding; however, only a minority of these patients received further imaging after skeletal maturity to evaluate the morphology of the developed acetabulum.

Delayed growth rate of the acetabular roof with subsequent lower shape differentiation of the roof also seems to be associated with splinting and cast treatment and might be responsible for later forms of dysplasia [12, 25].

A study by Sarkissian et al. [26] emphasized the usefulness of a follow-up X‑ray examination in the case of unremarkable hip sonography or after successful conservative therapy. The study examined 115 children over a period of 4 years who had abnormal findings on infant hip sonography at the time of birth. All these 115 patients showed clinical as well as sonographic normalization of findings after conservative therapy. An additional pelvic X‑ray of these children was performed at 6 and 12 months after birth, which showed evidence of residual DDH in 17% of these patients, despite the normal clinical and sonographic findings.

## Conclusion

Hip dysplasia is a complex, divergent and multifactorial disease that may present in different stages of life based on the severity of the underlying pathomorphology. The U3 ultrasonography examination that was introduced in Germany for universal DDH screening in 1996 has effectively reduced surgical interventions in the early years and nearly eliminated the entity of neglected hip dislocation. With increasing awareness in the area of hip preservation in young adults, it is becoming clear that milder forms of dysplasia are a frequent cause of hip pain in young adults. This study underlines that acetabular undercoverage cannot be ruled out based on a normal finding of ultrasonography screening. Furthermore, the study also showed that residual dysplasia may persist despite conservative treatment. It is therefore encouraged to perform a follow-up pelvic X‑ray after skeletal maturity for hips that had undergone conservative treatment. Furthermore, every symptomatic hip in young adulthood must be considered at risk of being dysplastic until proven otherwise and referred for further evaluation by an adult hip specialist.

## Abbreviations

AC: Anterior coverage
AI: Acetabular index
AP: Anteroposterior
DDH: Developmental dysplasia of the hip
EI: Femoral head extrusion index
LCEA: Lateral central edge angle
PAO: Periacetabular osteotomy
PC: Posterior coverage
U3: Postnatal infant examination at 3–8 weeks

## Declarations

All procedures performed in studies involving human participants or on human tissue were in accordance with the ethical standards of the institutional and/or national research committee and with the 1975 Declaration of Helsinki and its later amendments or comparable ethical standards. The study was approved by the local ethics committee of the Hannover Medical School (code: 10405_BO_K_2022). Informed consent was obtained from all individual participants included in the study.
